# Feedforward and feedback processes in vision

**DOI:** 10.3389/fpsyg.2015.00279

**Published:** 2015-03-12

**Authors:** Hulusi Kafaligonul, Bruno G. Breitmeyer, Haluk Öğmen

**Affiliations:** ^1^National Magnetic Resonance Research Center (UMRAM), Bilkent UniversityAnkara, Turkey; ^2^Department of Psychology, University of HoustonHouston, TX, USA; ^3^Center for Neuro-Engineering and Cognitive Science, University of HoustonHouston, TX, USA; ^4^Department of Electrical and Computer Engineering, University of HoustonHouston, TX, USA

**Keywords:** vision, visual system, feedforward, feedback, mechanisms

Hierarchical processing is key to understanding vision. The visual system consists of hierarchically organized distinct anatomical areas functionally specialized for processing different aspects of a visual object (Felleman and Van Essen, 1991). These visual areas are interconnected through ascending feedforward projections, descending feedback projections, and projections from neural structures at the same hierarchical level (Lamme et al., 1998). Even though accumulating evidence suggests that these three projections play fundamentally different roles in perception, their distinct functional roles in visual processing are still subject to debate (Lamme and Roelfsema, 2000). The focus of this Research Topic was the roles of feedforward and feedback projections in vision. In fact, our motivation to edit this Research Topic was threefold: (i) to provide current views on the functional roles of feedforward and feedback projections for the perception of specific visual features, (ii) to invite recent views on how these functional roles contribute to the distinct modes of visual processing, (iii) to provide recent methodological views to identify distinct functional roles of feedforward and feedback projections and corresponding neural signatures. As summarized below, these aims are largely achieved thanks to fourteen contributions to this issue.

## Feedforward and feedback projections for different aspects of a visual object

The cortical areas and the way they connect with each other lead to distinct pathways functionally specialized for processing different aspects of a visual object (Van Essen and Gallant, 1994). For example, the ventral processing stream has been associated with object recognition and identification. Romeo and Supèr (2014) have constructed a feedforward spiking hierarchical model for simulating IT cortex along the ventral stream. The simulation results indicate that figure-ground segregation occurs at an earlier level of processing relative to the level at which shape selection takes place. Wyatte et al. (2014) propose that object recognition requires more than feedforward processing. By reviewing a number of studies, they first differentiate two types of additional processing along the ventral stream: (i) early, short-distance (local) recurrent processes, and (ii) late, long-distance feedback processes related to attention. They further propose that early local recurrent feedback plays a functionally distinct role in attention-independent stimulus disambiguation, since it facilitates object recognition well before the onset of any attentional influences. Wutz and Melcher (2014) provide a review on temporal window for object recognition and individuation. They propose that mid-level vision adopts a temporal window whose duration is short enough for picking out separate objects (without appreciable smearing of their retinal images when they move), while simultaneously being long enough to integrate sufficient sensory information for accurate detection. Based on psychophysical and neurophysiological data, they suggest that phase synchronization plays a key role in this process by coordinating feedforward and feedback involved in complex and dynamic visual scenes. Several studies in this collection emphasize the role of feedback projections at different levels of processing within the ventral stream. Layton et al. (2014) propose a dynamic hierarchical model which can effectively perform figure-ground segregation in visual scenes with multiple objects. Their results indicate that the inhibitory feedback sharpens the population activity in the “lower stage” and that the dynamic balancing of feedforward signals with specific feedback mechanisms is crucial to identifying figural region. Furthermore, Layher et al. (2014) describe a model architecture to investigate the role of feedback mechanisms in learning new categories of visual objects. They basically use two types of feedback mechanisms to achieve seamless and automatic acquisition of category representation by an unsupervised learning mechanism integrated into a recurrent network architecture. Hence, they not only address the classic stability/plasticity dilemma but also elucidate how the predictive power of feedback mechanisms together with the feedforward sweep realize associative memory. Contour integration has been considered to be another crucial stage of visual object recognition. By varying the inter-element properties in a perceptual fading paradigm, Strother and Alferov (2014) focus on the individual roles of bottom-up feedforward and top-down feedback processing in such integration. In agreement with previous reports, their findings highlight the importance of feedforward processes in primary visual cortex (V1) and shape-related feedback from higher-tier visual cortical areas for contour integration.

## Roles of feedforward and feedback projections in different modes of visual processing

Accumulating evidence from modeling and experimental studies indicates that feedforward and feedback projections play important roles in different modes of visual processing and attention. However, their distinct contributions are still controversial. Khorsand et al. (2015) set the stage for feedforward and feedback contributions to the exogenous attentional selection. Bottom-up exogenous attention has been considered to rely only on feedforward processing of the external inputs. However, Khorsand et al. (2015) review recent experimental and theoretical studies supporting the view that stimulus dependent processing involves feedback connections and signals running in top-down direction of the hierarchy as well. Their review raises an important conceptual issue and provides an account of feedforward and feedback contributions to exogenous attentional shifts. In another study, Rensink (2014) identifies different levels of processing for iconic memory by using a modified visual search paradigm. Besides feedforward processing, he highlights the importance of two types of feedback projections (due to horizontal connections within a level as well as links between different levels) for iconic memory. He further characterizes “iconic,” “preattentive,” and “attentive” representations within this framework. As briefly mentioned above, based on the literature about visual object recognition, Wyatte et al. (2014) dissociate the late top-down processing originating from frontoparietal areas from early recurrent local projections within the ventral processing stream. They also review some studies emphasizing that this late top-down processing to striate cortex provides attentional support for salient or behaviorally-relevant features.

## Explaining various visual phenomena by feedforward and feedback processes

The notions of feedforward and feedback processing have been extensively used to explain various visual phenomena. Di Lollo et al. (2013) hypothesizes that reentrant (feedback) processing gives the best account for a form of visual masking called object substitution masking (OSM). On the other hand, Põder et al. (2014) presents the contrasting view that reentrant processing is not necessary to explain OSM and that the attentional gating model is the simplest and most reasonable explanation for OSM results. Silverstein (2015) takes an interesting approach to examine the roles of feedforward and feedback processes in visual backward masking. Using a biophysical model of V1 and V2, he explains visual processing in terms of interacting cortical attractors. The simulation results indicate that both feedforward and feedback processes predict several aspects of backward masking. Additionally, Petro et al. (2014) focus on the functional role of cortical feedback projections on V1. By reviewing the most current theory and experimental data, they discuss how top-down feedback signals conveying information from higher-processing stages (e.g., prediction, reward, memory and behavioral context) are involved in shaping sensory processing in V1 and hence, explain recent experimental findings along this direction.

A contrasting view is provided by Clarke et al. (2014), who argue against the usefulness of making feedforward and feedback distinctions for explaining experimental results. They tested three existing models with different local/global and feedback/feedforward characteristics to see whether they can account for some recent findings on visual crowding. All three models failed to predict the results even qualitatively. Clarke et al. (2014) discuss these model failures within the context of a broader view and suggest that the dichotomies such as feedforward/feedback and local/global may not be useful for scientists designing experiments to understand vision. Bachmann (2014) argues another interesting point. He basically posits that experimental findings that have been proposed to support models of specific top-down re-entrant processing could equally support those with a generic, non-specific feedback loop.

Taken together, the research topic presents a timely addition to the field of vision research and to understanding the functional principles of brain in general. It provides an update on the roles of feedforward and feedback projections in several but not all types of visual processing. For example, an update about the roles of feedforward and feedback projections in motion processing (mostly carried out by the dorsal pathway) is missing. The advent of optogenetics and neuroimaging has provided additional remarkable investigative tools. How these recent techniques will contribute to the prevailing arguments of feedforward and feedback projections in vision is still open. We hope this issue will inspire the readers and act as a catalyst for future work on the issues of feedforward and feedback processes in vision.

### Conflict of interest statement

The authors declare that the research was conducted in the absence of any commercial or financial relationships that could be construed as a potential conflict of interest.
